# The impact of mortality salience and explicit self-esteem on plastic reduction intention: A moderated mediation model

**DOI:** 10.1371/journal.pone.0320059

**Published:** 2025-03-24

**Authors:** Hsiao-Ling Chiu, Kung-Jeng Wang, Tsang-Hsien Wang

**Affiliations:** Graduate Institute of Management, National Taiwan University of Science and Technology, Taipei, TaiwanROC; Jordan University of Science and Technology, JORDAN

## Abstract

Mortality salience (MS) and self-esteem affect attitudes and behavioral intentions in various contexts, including pro-environment behavioral intentions. This study aimed to explore how MS and explicit self-esteem impact on attitude towards plastic reduction for future generations (APRFG) and intention to reduce plastic use, by integrating the Theory of Planned Behavior (TPB) and Terror Management Theory (TMT). An experimental design was employed, with 357 participants aged 20 to 70 recruited via an online platform. The participants were randomly assigned to either an MS condition, or a control condition and then measured their explicit self-esteem by the Rosenberg Self-Esteem Scale. Subsequently, the experimental group was exposed to information on the fatal health risks associated with microplastics, while the control group was reminded of their own experiences of dental pain. After a few minutes, all participants were asked to complete a self-report questionnaire, including APRFG, subjective norm, perceived behavioral control, and intention to reduce plastic use. Data were analyzed using the PROCESS macro to examine mediation and moderation effects. Our findings showed that MS significantly enhanced APRFG, subjective norm, and perceived behavioral control, which in turn led to stronger intention to reduce plastic use. Among these three factors, the mediating effect of APRFG was the most pronounced, highlighting its central role in linking MS to plastic reduction intention. MS, when combined with explicit self-esteem, significantly influences APRFG and intention to reduce plastic use. The relationships between MS and APRFG/plastic reduction intention were particularly pronounced among the participants with lower explicit self-esteem. Policymakers can leverage these insights to craft marketing messages that enhance plastic-reduction efforts, taking into account individual differences in explicit self-esteem.

## 1. Introduction

Plastic waste poses significant environmental and human health risks [1–5]. Despite extensive government efforts to limit plastic usage through policies and regulations [3,6,7], the amount of plastic waste is expected to continue growing, exacerbating these risks [8]. This underscores the necessity for the public to actively engage in reducing plastic usage to effectively address this issue [9]. Consequently, understanding the determinants of pro-environmental behaviors, particularly those related to plastic reduction, is a critical topic in social psychology [10,11].

From a social psychology perspective, enhancing consumers’ sustainable intentions can strengthen their sustainable behaviors [12]. According to the theory of planned behavior (TPB) [12], individuals’ beliefs drive their attitudes and intentions in various contexts. For instance, materialistic beliefs weaken green consumption attitude, thereby reducing green consumption intention [13]. However, environmental stimuli, such as death-related messages, can trigger materialistic beliefs and affect environmentally green concern [14]. Therefore, exploring if health risks or death-related concerns associated with plastic pollution can enhance attitude and intention towards plastic reduction is a crucial step for developing effective communication strategies targeted at the public. However, existing studies showed that the direct relationship between death-related stimuli and sustainable or green attitudes is not significant without considering mediating or moderating variables [14–16]. This suggests policymakers should consider these factors when promoting environmental initiatives by emphasizing plastic health risks.

Terror Management Theory (TMT) [17] provides a theoretical foundation for examining the manipulation of death induced messages on the health risks of plastics. TMT posits that the awareness of death from the stimuli in the environment makes individuals aware of their inevitable mortality, known as mortality salience (MS). To reduce the anxiety and negative emotions engendered by MS [18], individuals initially adopt proximal defense strategies to suppress these emotions, making them unconscious [19]. However, this leads to death-thoughts entering the unconscious state, inducing distal defense strategies to alleviate unconscious death anxiety. The choice of distal defense strategy depends on what makes the individual feel more valuable and aligned with their worldview [19]. According to TMT research, MS prompts individuals to place greater value on future generations, as they serve as a form of distal defense mechanism against the fear of death [20–23]. By having offspring, individuals ensure the continuation of their genes, legacy, culture, and humanity, which helps them cope with the existential threat of mortality. Research even shows that MS can increase people’s selfless care and commitment to future generations [24–25]. Following this logic, this study suggests that MS strengthens people’s efforts to support future generations, such as reducing plastic use for the sake of their health. This emphasis helps enhance individuals’ self-worth, thereby reducing existential anxiety. Consequently, MS affects attitudes and intentions across various contexts, depending on how individuals respond to these death-related stimuli. More importantly, individuals with high or low explicit self-esteem exhibit different distal defense strategies in response to MS, demonstrating the anxiety-buffering role of explicit self-esteem: explicit self-esteem moderates the relationship between mortality salience stimuli and distal defense behavior [19,26]. From TMT perspective, we understand that if a plastic reduction worldview is preferred, explicit self-esteem will interact with MS to influence attitude towards plastic reduction for the benefit of future generations, as well as intentions to reduce plastic use.

This study is aimed at exploring the impact of MS induced by plasticizers on attitude towards plastic reduction for future generations (APRFG) and intention to reduce plastic use, employing an experimental method grounded in the TPB and TMT. Furthermore, this study examines whether lower explicit self-esteem strengthens APRFG and intention to reduce plastic use in the context of MS. Additionally, it assesses the impact of MS on subjective norm and perceived behavioral control related to plastic reduction [12].

The objectives of this study are as follows: Firstly, to determine whether manipulating environmental death stimuli, such as MS induced by the health risks of plastics, enhances APRFG and intention to reduce plastic use. Secondly, to assess whether MS caused by the health risks of plastics increases subjective norm and perceived behavioral control regarding plastic reduction, thereby affecting intention to reduce plastic use. Thirdly, to investigate whether individuals with various explicit self-esteem exhibit different levels of APRFG and intention to reduce plastic use after exposure to MS engendered by health risks of plastics. Fourthly, to examine whether there are differences in the relative impact of APRFG, subjective norm, and perceived behavioral control on intention to reduce plastic use.

## 2. Theoretical background and hypotheses

### 2.1. Mortality salience, attitude towards plastic reduction for future generations, intention to reduce plastic use

When facing mortality salience (MS), individuals engage in various consumption behaviors to alleviate their sense of mortality, including domestic, indulgent, impulsive, materialistic, experiential, innovative, nostalgic, hedonic, and prosocial consumption [27]. These behaviors are driven by personal preferences and worldviews, with materialism often being self-centered and negatively associated with pro-environmental attitudes and behaviors [13,28]. However, the desire for offspring, intensified by the awareness of death, underscores the longing for symbolic immortality [20–23]. Subsequent studies have also confirmed that MS can enhance people’s selfless care and commitment toward future generations [24–25]. Recently, a study from China also indicated that MS can trigger stronger descendant continuity [23]. Since the desire for offspring and considerations for the welfare of future generations can serve as an anxiety-buffering worldview defense strategy [21,23–25], and MS can also enhance green concern [14], this study suggest that attitude towards plastic reduction aimed at safeguarding the health of future generations may also serve as a defense mechanism for self-preference. In fact, other studies have found that appeals to children are associated with enhanced pro-environmental behaviors [29]. Although reducing plastic use is not entirely equivalent to green consumption or green concern, Khan et al. [30] found a moderate to high correlation (standardized regression coefficient =  0.42) between plastic ban policies and green consumer behavior, indicating a potential link between plastic reduction and green consumption. Therefore, this study suggests that reducing plastic use is likely related to green consumption [31] or green concern [14]. Based on these studies, this research aims to use an experimental method to explore whether MS has a positive influence on the intention to reduce plastic use and on attitude towards plastic reduction for future generations (APRFG).

H1 MS is positively related to individuals’ intention to reduce plastic use.
H2 MS is positively related to APRFG.

### 2.2. Mortality salience as an implicit intervention for planned behavior

The Theory of Planned Behavior (TPB), introduced by Icek Ajzen in 1985 [32], posits that human behavior is influenced by three primary factors: behavioral beliefs, normative beliefs, and control beliefs. These shape an individual’s attitudes towards the behaviors, subjective norms, and perceived behavioral control, which together determine behavioral intentions. Attitudes reflect an individual’s evaluation of performing a behavior, based on beliefs about outcomes and their desirability. Subjective norms refer to perceived social pressure, influenced by others’ expectations and the motivation to comply with them. Perceived behavioral control is the perceived ease or difficulty of performing the behavior, based on the presence of facilitating or hindering factors and their impact. Together, these factors collectively form the behavioral intention, indicating an individual’s readiness to engage in the behavior. A favorable attitude, stronger subjective norm, and greater perceived behavioral control lead to a stronger intention to engage in the behavior [33–34]. TPB is an extensively validated theoretical framework frequently employed to investigate various pro-environmental behaviors, including plastic reduction [35–36]. Consistent with prior findings supporting TPB in the context of plastic use [9,10,35,36], this study suggests that attitude towards plastic reduction for future generations, subjective norm, and perceived behavioral control are associated with intention to reduce plastic use.

This study integrates Terror Management Theory (TMT) and the Theory of Planned Behavior (TPB) to investigate plastic reduction behavior, similar to how previous research has connected other theories to understand green consumption behavior [37]. While TPB traditionally predicts plastic reduction intention through attitude, subjective norm, and perceived behavioral control [1,10], existing studies suggest incorporating additional factors into the TPB framework to better understand intentions to engage in green consumption [11,37,38]. For instance, environmental concern influences attitudes towards plastic use and green product consumption, as well as subjective norm and perceived behavioral control [11,37]. Perceived consumer effectiveness strengthens green consumption attitude [39], and environmental risk perception is linked to plastic reduction behavior [35]. Conversely, materialistic beliefs weaken green consumption attitude and intention [13]. In TMT studies, MS enhances environmental concern [14], which reshapes individuals’ attitude towards sustainability, as well as their subjective norm and perceived behavioral control [11,37], ultimately influencing their intention to reduce plastic use and engagement with green consumption [11,37]. Therefore, this study posits that MS, considered as a precursor to environmental concern, may influence intention to reduce plastic, with this relationship mediated by attitude towards plastic reduction for future generations, subjective norm and perceived behavioral control.

However, these studies highlight several issues. First, most self-reported surveys cannot explore causal relationships affecting the four TPB variables as effectively as an experimental method. For example, Truelove et al. [40] used an experimental approach to study the impact of pledge interventions on attitude, subjective norm, perceived behavioral control, and intention towards single-use plastic reduction, yielding significant results. Second, studies have shown that explicit self-esteem and MS influence materialism, which in turn affects green consumption attitude [14,41]. MS also interacts with personality traits (e.g., environmental contingency of self-worth, political orientation) to impact environmental concern [15,42], which in turn influences green consumption attitude, subjective norm, and perceived behavioral control [37]. Thus, MS and explicit self-esteem are upstream variables impacting attitude, subjective norm, and perceived behavioral control. Further investigation is needed to understand their effects on plastic reduction intention. Third, MS and explicit self-esteem implicitly influence various consumption attitudes and intentions [27]. Understanding how MS and explicit self-esteem predict attitudes, subjective norms, and perceived behavioral control is crucial for grasping the unconscious factors affecting green consumption intentions. Therefore, this study employs an experimental method to examine the impact of MS on APRFG, subjective norm, perceived behavioral control, and behavioral intention to reduce plastic use. Based on the TPB and the extant literature, we propose the following hypotheses:

H3 MS is positively related to subjective norm.
H4 MS is positively related to perceived behavioral control.
H5 APRFG is positively related to individuals’ intention to reduce plastic use.
H6 Subjective norm is positively related to individuals’ intention to reduce plastic use.
H7 Perceived behavioral control is positively related to individuals’ intention to reduce plastic use.
H8 APRFG mediates the relationship between MS and individuals’ intention to reduce plastic use.
H9 Subjective norm mediates the relationship between MS and individuals’ intention to reduce plastic use.
H10 Perceived behavioral control mediates the relationship between MS and individuals’ intention to reduce plastic use.

### 2.3. Explicit Self-esteem, attitude towards plastic reduction for future generations, and intention to reduce plastics use

Another area of interest in this study is the relationship between explicit self-esteem and APRFG. Explicit self-esteem is defined as the deliberate and conscious evaluation of oneself [41]. The Rosenberg Self-Esteem Scale (RSE) [43] is the most commonly used measure for explicit self-esteem. Previous research has shown that explicit self-esteem is associated with stronger materialistic values. Building on the TMT studies [27], explicit self-esteem often interacts with MS, influencing consumer behaviors. If green consumption is viewed positively, it can enhance explicit self-esteem and help reduce anxiety induced by MS. For instance, Rahimah et al. [14] found that individuals with higher explicit self-esteem exhibited greater materialism as a means to counteract the effects of MS, which in turn enhanced their green attitudes. However, if green consumption is not viewed positively, individuals may distance themselves from behaviors or products that threaten their self-identity and explicit self-esteem. For example, Johnstone and Tan [31] found negative perceptions of environmental behaviors or green consumption through qualitative interviews. Some people might perceive that buying green products or engaging in environmental activities could lead to being viewed as odd, radical, or hypocritical, due to societal stereotypes and misunderstandings about environmentalists and green products. This reluctance to publicly support or engage in green consumption can be detrimental to those who place high value on their explicit self-esteem. These studies highlight the varying impacts of explicit self-esteem on attitude towards plastic reduction.

Therefore, through an experimental method, we aimed to explore whether MS and explicit self-esteem interact to influence APRFG and intention to reduce plastic use. Based on the notion that MS promotes a desire for offspring to continue one’s legacy [20–23], we hypothesize that individuals with lower explicit self-esteem, due to their lack of confidence, may be more likely to adopt this worldview defense strategy in response to death anxiety. This would manifest as a more proactive attitude towards plastic reduction for the benefit of future generations and a greater intention to reduce plastic use. Hence, we propose the following hypotheses:

H11 Explicit Self-esteem moderates the effect of MS on APRFG.
H12 Explicit Self-esteem moderates the indirect association between MS and individuals’ intention to reduce plastic use via APRFG, such that the mortality salience-attitude pathway would be stronger in group with lower explicit self-esteem.

**Fig 1:**
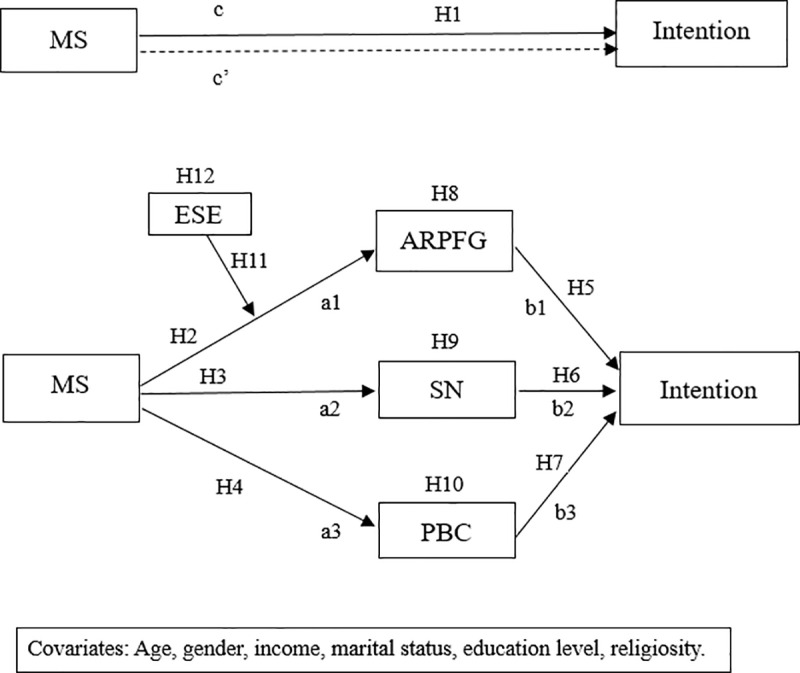
Conceptual model. MS =  Mortality salience; APRFG =  Attitude towards plastic reduction for future generations; SN =  Subjective norm; PBC =  Perceived behavioral control; Intention =  Intention to reduce plastic use; ESE =  Explicit self-esteem.

Given that we suppose explicit self-esteem only moderates the first stage of the mediation path, this present study would refer to it as a first-stage moderation model. Based on the above hypotheses, our study proposes the conceptual model and presented it in Fig 1.

## 3. Materials and methods

### 3.1. Study design and data collection

#### 3.1.1. Participants.

This study was approved by the Research Ethics Committee of National Taiwan University. The experiment was conducted through the online platform dosurvey.com.tw, using a random sample of 600 participants, aged 20 to 70, from various regions across Taiwan. To begin with, dosurvey.com.tw obtained informed consent from each participant for this anonymous survey. Then participants were randomly assigned to either an MS condition or a control condition, with 300 participants in each group. A total of 85% of the experimental group (255) and 83% of the control group (249) agreed to complete the survey in exchange for an NT$100 gift voucher offered by the platform. For those willing to participate, we administered the Generalized Anxiety Disorder 7-item scale (GAD-7), a self-reported measure used to assess anxiety symptoms [44]. Participants with severe anxiety were excluded from the study (32 from the experimental group and 29 from the control group). Additionally, participants with incomplete answers or who took less than 8 minutes to complete the survey were excluded (41 from the experimental group and 45 from the control group). The final valid sample consisted of 357 participants, with 182 in the experimental group and 175 in the control group. The survey was conducted in Nov 2024. The participants in the experimental group and control group spent an average of 14 minutes and 12 minutes, respectively, completing all the survey items. This included demographic questions, experimental tasks, and the assessment scales relevant to the study.

For the sample size rationale, since there is no identical study to reference, we referred to Qi [23] for TMT research on Chinese participants (specifically the impact of MS on having offspring). Given the cultural similarity between Taiwanese participants in our study and Chinese participants in Qi’s [23] study, we adopted the minimum required sample size of 272 (α =  0.05, power =  0.95) as calculated by Qi [23]. Additionally, since attitude towards plastic reduction are related to green concern, we also referenced Rahimah et al. [14], which reported an R² = .53 (f² =  1.13) with religiosity moderating the relationship between mortality salience/ESE and green concern. Based on Rahimah et al. [14], a minimum sample size of 36 was determined using power analysis with G * Power 3.1.9.7 [45] for multiple linear regression (α =  0.05, power =  0.95). Therefore, we chose the stricter minimum sample size of 272 for this study. Our final sample size of 357 is 31% above the minimum.

#### 3.1.2. Explicit self-esteem.

Subsequently, all participants were asked to complete Rosenberg self-esteem scale [43] (10 items, 7-point Likert scale), which is considered a measure of explicit self-esteem [41]. We adjusted the scores of the reverse-coded items to their corresponding positive scores, with higher scores indicating higher levels of explicit self-esteem. The 10-item scale had a Cronbach’s alpha coefficient of 0.89. The score of the 10 items were summed and averaged to represent the explicit self-esteem score.

#### 3.1.3. Mortality salience.

After measuring explicit self-esteem, the experimental group was exposed to a text description of the health risks of microplastics to humans, along with two open-ended questions [46] to induce MS. The control group utilized the commonly used pain from toothache experiences in TMT experiments [19], which evokes non-death-related discomfort (S1 Appendix). The experimental and control groups were represented by dummy variables 1 and -1, respectively, for statistical analysis. The MS manipulation text is as follows: “Microplastics are plastic particles smaller than 5 millimeters, formed from plastic waste in the environment. Items like discarded plastic containers, plastic tableware, plastic bags, and straws break down over time through sunlight exposure, becoming brittle, decomposing, and fragmenting into microplastics. In the ocean, these microplastics can accumulate in marine organisms through contact or ingestion. Humans can easily ingest, drink, or inhale these contaminated organisms, water, or air. A study published in the Proceedings of the National Academy of Sciences reports that each liter of bottled water contains up to 240,000 microplastic particles from seven different types of plastic. Additionally, research in the New England Journal of Medicine indicates that people with microplastics in their carotid artery tissues have more than four times the risk of heart disease, stroke, and death within three years compared to those without microplastics [47]. It is unimaginable that our daily diet could be contaminated by microplastics, harming our health without us realizing it. What will happen to us as we physically die? What emotions that the thought of our own death arouses [46]?”

#### 3.1.4. Delay.

All participants then completed a standard delay and distraction task lasting approximately 5–6 minutes, commonly used in terror management theory research. This task involved reading a brief, neutral passage and answering related questions [19]. A delay is necessary because TMT posits that unconscious defenses against death only activate after sufficient time has elapsed to push death thoughts into the unconscious [19]. Consequently, standard procedures for examining MS effects include a delay between death thought activation and behavior assessment. For this reason, as a delay task, the experimental group read a passage excerpted from an article regarding artificial intelligence [48] after responding to the MS-related open-ended questions. Conversely, the control group, after being informed about the experiences of dental pain, read the same article immediately without any MS-related questions.

#### 3.1.5. Self-report questionnaire.

At last, the participants were asked to complete a self-report questionnaire, including the measures of APRFG, subjective norm, perceived behavioral control, and intention to reduce plastic use (Table 1). The measuring items were adapted from Hsu et al. [49] and modified these items to suit the conditions of reducing plastic use. And the measuring items were assessed using a 7-point Likert scale. The self-report questionnaire also collected socio-demographic characteristics of the participants, including age, gender, income, marital status, education level, religiosity.

**Table 1:** Measurement items.

variables	Measurement items
Attitude towards plastic reduction for future generations	Reducing the use of plastic products is good for the next generation.
	Reducing the use of plastic products is positive for the next generation.
	Reducing the use of plastic products contributes to the health of the next generation.
Subjective norm	Most of the people important to me agree that plastic use should be reduced.
	Most of the people I care about agree that plastic use should be reduced.
	My family and friends agree that plastic use should be reduced.
Perceived behavioral control	If I want to reduce the use of plastic products, I can do it.
	I have enough money to choose non-plastic products (like glass bottles).
	I have enough time to choose non-plastic products (like glass bottles).
Intention to reduce plastic use	I will reduce the use of plastic tableware.
	I will reduce the use of plastic bags.
	I will reduce the use of plastic bottled water.
	I will reduce the use of plastic straws.
	I will reduce the use of plastic products in my daily life.

### 3.2. Statistical analyses

This study first used SPSS AMOS 26 to conduct Confirmatory Factor Analysis (CFA) to validate the measurement model among variables, including APRFG, subjective norm, perceived behavioral control, and intention to reduce plastic use. Subsequently, we tested the hypotheses (H1-H7) and mediation effects (H8-H10) using MODEL 4 in PROCESS macro [50–51] with 5000 bootstrapped samples, controlling for six variables: age, gender, income, marital status, education level, religiosity. Following Baron and Kenny’s [52] recommendations, mediation requires a significant effect of the independent variable on the dependent variable, and a linear relationship between the independent variable and the mediator. The PROCESS macro, developed by Hayes, is a robust statistical tool for analyzing mediation, moderation, and conditional process models [50–51]. It employs a nonparametric percentile bootstrap method for robust effect estimation without assuming normality. The PROCESS macro by Hayes offers a range of pre-specified models, each designed to assess various types of mediation and moderation effects. In this study, MODEL 4 in PROCESS macro was selected for parallel mediation analysis, allowing simultaneous estimation of the unique contributions of multiple mediators (APRFG, subjective norm, and perceived behavioral control) in linking MS to intention to reduce plastic use. Finally, we utilized MODEL 7 in PROCESS macro [50–51] to examine the moderation effect (H11) and moderated mediation effect (H12), focusing on the moderating role of explicit self-esteem. MODEL 7 in PROCESS macro is designed to test if a moderator influences the strength of a mediation pathway, allowing for the analysis of conditional indirect effects. Therefore, it is appropriate for examining the moderation effect (H11) and moderated mediation effect (H12) in this study.

### 3.3. Demographic characteristics

The randomly sampled participants had an average age of 40 to 50 years, with women comprising 51% of the sample. The age distribution closely mirrored the population structure of Taiwan. Additionally, 82% of the participants held a college degree or higher, and 57% were married (Table 2).

**Table 2:** Demographic characteristics.

Characteristics		Frequency	Percentage
Gender	Male	175	49%
	Female	182	51%
Age	21-30	52	15%
	31-40	81	23%
	41-50	87	24%
	51-60	87	24%
	61-70	50	14%
Education Level	Senior high school or below	63	18%
	College or bachelor’s degree	238	66%
	Master’s degree or above	56	16%
Annual Income	No income	23	6%
	Below USD 16,150	111	31%
	USD 16,150 – 32,200	153	43%
	USD 32,201– 48,380	51	14%
	USD 48,381– 80,645	12	4%
	USD 80,646 – 161,270	3	1%
	USD 161,270 above	4	1%
Marital status	Single	152	43%
	Married	205	57%
Religiosity	No faith	141	39%
	Has faith, but not devout	126	35%
	Devout	88	25%
	Very devout	2	1%
Mortality Salience	Experimental group	182	51%
	Control group	175	49%

## 4. Results

### 4.1. Reliability and discriminant validity

We first conducted CFA using SPSS AMOS 26 to assess the fit among the four dimensions, including the mediators and dependent variable. The results indicated that the factor loadings on each latent variable ranged from 0.75 to 0.90 (Table 3). The goodness-of-fit indices of the measurement model demonstrated an acceptable fit: χ²/df =  1.86 ( < 3), RMSEA =  0.049 ( < 0.08), GFI =  0.950 ( > 0.90), CFI =  0.983 ( > 0.90), IFI =  0.983 ( > 0.90), TLI =  0.979 ( > 0.90), SRMR =  0.040 ( < 0.08) [53]. Cutoff values for these indices were suggested by Hu and Bentler [53], West et al. [54] and Doronin et al. [55]. For all variables, the values of Cronbach’s alpha, McDonald’s omega coefficients (ω) and composite reliability (CR) exceeded 0.80, and the average variance extracted (AVE) values were greater than 0.6. The square root of the average variance extracted (AVE) for ARPFG, subjective norm, perceived behavioral control and intention to reduce plastic use exceeded the correlation coefficient between them. Table 4 showed the correlations among the variables, including the independent variables. The absolute values of skewness and kurtosis for the mediator and dependent variables were all less than 1, indicating that their distributions were close to normal. The mediator and dependent variables also exhibited good discriminant validity.

**Table 3:** Measurements.

	Indicators	Mean	Factor loading	Cronbach’s alpha	McDonald’s omega coefficients	CR	AVE
ARPFG	AR1	6.06	0.90	0.92	0.95	0.92	0.79
	AR2	5.98	0.90				
	AR3	5.94	0.87				
SN	SN1	5.20	0.88	0.89	0.93	0.89	0.72
	SN2	5.12	0.84				
	SN3	5.22	0.83				
PBC	PB1	5.65	0.75	0.84	0.91	0.85	0.65
	PB2	5.27	0.83				
	PB3	5.28	0.84				
Intention	IN1	5.79	0.88	0.92	0.94	0.92	0.71
	IN2	5.73	0.86				
	IN3	5.60	0.79				
	IN4	5.66	0.86				
	IN5	5.68	0.82				

Goodness-of-fit indices (N =  357): χ²/df =  1.86; RMSEA =  0.049; GFI =  0.950; CFI =  0.983; IFI =  0.983; TLI =  0.979; SRMR =  0.040.

APRFG =  Attitude towards plastic reduction for future generations; SN =  Subjective norm; PBC =  Perceived behavioral control; Intention =  Intention to reduce plastic use.

**Table 4:** Correlation analysis and discriminant validity (N = 357).

	Mean	SD	1	2	3	4	5	6	7	8	9	10	11	12
1.MS			1											
2.ESE	4.84	.87	.06	1										
3.APRFG	5.99	.86	.28^***^	.22^**^	.89									
4.SN	5.18	.89	.24^***^	.28^***^	.35^***^	.85								
5.PBC	5.40	.92	.20^***^	.42^***^	.43^***^	.49^***^	.81							
6.Intention	5.69	.90	.27^***^	.30^***^	.70^***^	.46^***^	.56^***^	.84						
7.Age	3.01	1.27	−.04	.23^***^	.10	.13^*^	.11^*^	.17^***^	1					
8.Gender			−.04	.05	−.12^*^	−.05	−.01	−.19^***^	.14^*^	1				
9.Marital status			−.10	−.26^***^	−.05	−.06	−.08	−.13^*^	−.54^***^	−.09	1			
10.Income	2.84	1.04	.03	.20^**^	.09	.06	.21^***^	.05	.15^**^	.14^**^	−.22^**^	1		
11.Education	1.98	.58	.03	.18^***^	.08	.01	.02	.01	−.21^***^	.02	.11^*^	.23^***^	1	
12.Religiosity	1.86	.80	.04	.09	.03	.06	.12^*^	.03	.24^***^	−.01	−.14^*^	.10	−.10	1

Skewness and Kurtosis: ESE: -.18,.33; APRFG: -.80;.67; SN: -.23, -.02; PBC: -.49, -.22; Intention: -.75, -.80.

The square root of the average variance extracted (AVE) for ARPFG, SN, PBC or Intention exceeds the correlation coefficient between them.

Age, range from 1 to 5 (year-old of 21-30, 31-40, 41-50, 51-60, 61-70); Gender (male:1; female: -1); Marriage (single:1; married: -1); Income (annual income from low to high: 1-7); Education (education level from low to high: 1-3); Religiosity (No faith, Has faith but not devout, devout, very devout:1-4, respectively).

MS =  Mortality salience; ESE =  Explicit self-esteem; APRFG =  Attitude towards plastic reduction for future generations; SN =  Subjective norm; PBC =  Perceived behavioral control; Intention =  Intention to reduce plastic use.

Furthermore, we conducted the Breusch-Pagan test to assess potential heteroscedasticity in the effects of all independent variables on the two dependent variables, APRFG and intention to reduce plastic use. The results indicated that the p-values for the residuals in relation to nine variables excluding SN and PBC were greater than.05, suggesting no significant heteroscedasticity in the effects of those nine variables (MS, ESE, interaction effect of MS and ESE, six covariates) on these outcomes. However, we still employed heteroscedasticity-consistent standard errors (HC3) to address the model’s heteroscedasticity issue [56].

### 4.2. Hypothesis testing

To examine the effects of MS on the dependent and mediator variables, firstly, we used independent sample t-tests to test hypotheses H1-H4. The findings indicated significant differences between the experimental and control groups in the following variables: intention to reduce plastic use (M _Target_ =  5.93, SD = .87 vs. M _Control_ =  5.45, SD = .88, t =  5.23, p < .001, Cohen’s d =  0.55), APRFG (M _Target_ =  6.23, SD = .79 vs. M _Control_ =  5.75, SD = .86, t =  5.48, p < .001, Cohen’s d =  0.58), subjective norm (M _Target_ =  5.39, SD = .87 vs. M _Control_ =  4.96, SD = .85, t =  4.75, p < .001, Cohen’s d =  0.50), and perceived behavioral control (M _Target_ =  5.58, SD = .86 vs. M _Control_ =  5.21, SD = .94, t =  3.89, p < .001, Cohen’s d =  0.41). These results support hypotheses H1-H4.

Secondly, in addition to testing between-group differences, we used a dummy variable for MS and controlled for six variables. We then performed linear regression to test H1-H4 and re-examine H5-H7. The results in Table 5 showed significant positive linear relationships between the variables. Thirdly, we used MODEL 4 in SPSS PROCESS [50–51] to test the mediation effects. The analysis revealed that all three mediator variables fully mediated the relationship between MS and intention to reduce plastic use, supporting H8, H9, and H10. The indirect effects were B = .13, SE = .03, 95% CI [.0785,.1806]; B = .03, SE = .01, 95% CI [.0062,.0495]; B = .05, SE = .02, 95% CI [.0237,.0843]. Among these, the unstandardized regression coefficients (B) indicated that APRFG had the strongest indirect effect.

**Table 5:** The findings from parallel mediation model tests (unstandardized).

ARPFG (M1)										
Total effect (H1)		Direct effect		IV�M1(H2)		M1�DV(H5)		Indirect effect (H8)		
c		c’		a1		b1		a1xb1		95% CI
B	SE	B	SE	B	SE	B	SE	B	SE	
.24^***^	.05	.03	.04	.24^***^	.05	.53^***^	.05	.13	.03	**[.0785, .1806]**
**Subjective Norm (M2)**										
Total effect (H1)		Direct effect		IV�M2 (H3)		M2�DV(H6)		Indirect effect (H9)		
c		c’		a2		b2		a2xb2		95% CI
B	SE	B	SE	B	SE	B	SE	B	SE	
.24^***^	.05	.03	.04	.22^***^	.05	.12^*^	.05	.03	.01	**[.0062, .0495]**
**Perceived Behavioral Control (M3)**										
Total effect (H1)		Direct effect		IV�M3 (H4)		M3�DV(H7)		Indirect effect (H10)		
c		c’		a3		b3		a3xb3		95% CI
B	SE	B	SE	B	SE	B	SE	B	SE	
.24^***^	.05	.03	.04	.18^***^	.05	.28^***^	.05	.05	.02	**[.0237, .0843]**

IV: Independent variable (Mortality Salience); DV: Dependent variable (Intention to reduce plastic use); M1: the 1st mediator; M2: the 2nd mediator; M3: the 3rd mediator.

B, unstandardized regression coefficients; SE, standard error; LLCI, lower limit confidence interval; ULCI, upper limit confidence interval; Bolded confidence intervals (95%) do not include zero, indicating a significant indirect effect. ^*^p < .05; ^**^p < .01; ^***^p < .001.

Controlling for age, gender, marriage, income, education level, religiosity, variance explained by the models (*R*^2^) is .62.

**Fig 2:**
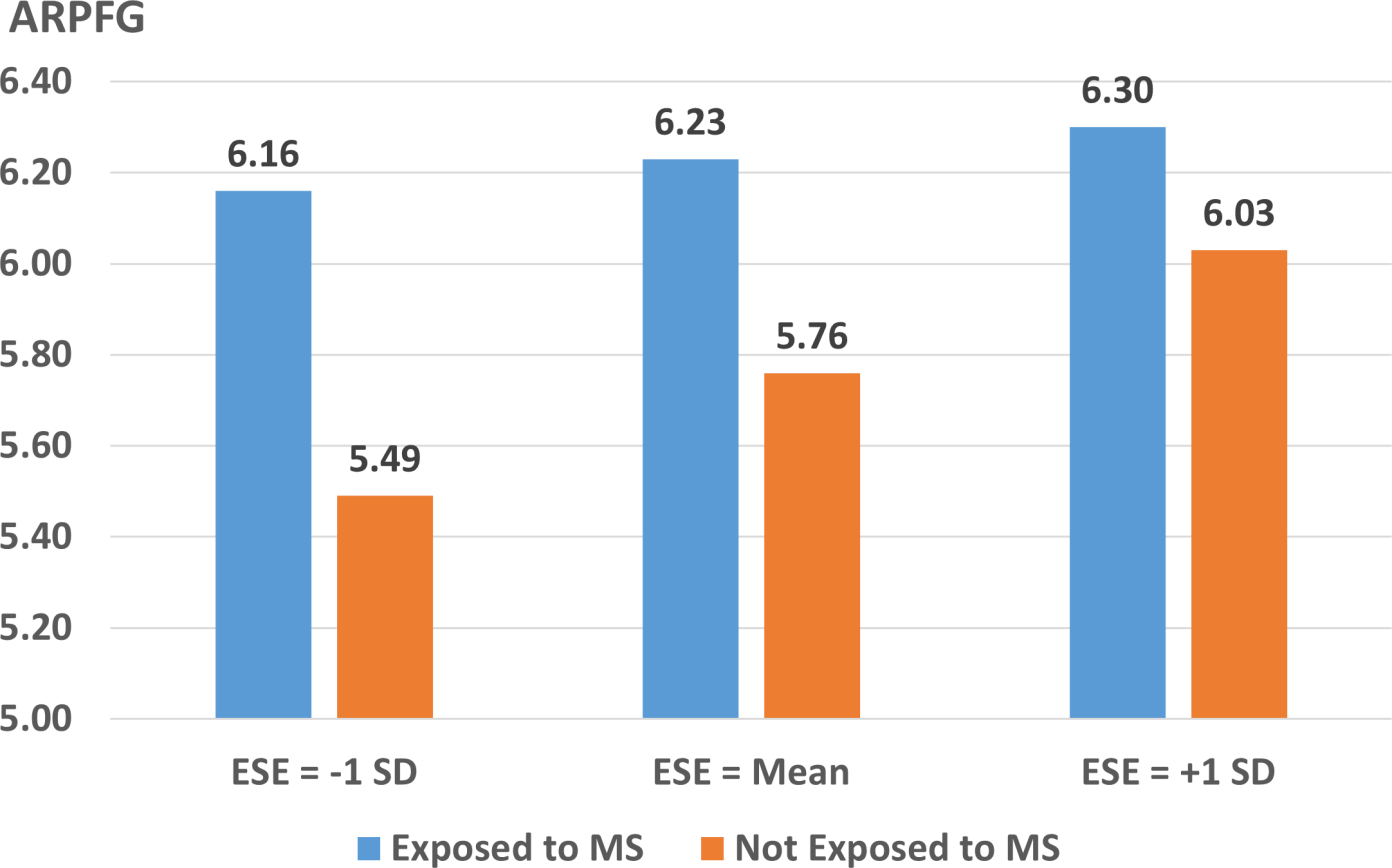
Moderating effect of ESE. MS =  Mortality salience; ESE =  Explicit self-esteem; APRFG =  Attitude towards plastic reduction for future generations.

Fourthly, we employed both hierarchical regression and MODEL 7 in SPSS PROCESS to analyze the interaction effect of explicit self-esteem and MS on APRFG. Table 6 showed a significant interaction effect (Beta =  -.12, p < .05, SE = .05), confirming H11. We further analyzed the APRFG under the influence of MS among the participants of different explicit self-esteem. Additionally, Table 7 and Fig 2 indicated that under three different levels of explicit self-esteem (-1SD, mean, + 1SD), MS significantly increases APRFG and the intention to reduce plastic use. However, this positive effect is particularly pronounced among participants with lower level of explicit self-esteem. The index of moderated mediation was -0.06, BootSE = .0241, 95% CI [-.1087, -.0125], confirming that explicit self-esteem served as a moderated mediation role in the overall model, supporting H12. The empirical results of the hypotheses are summarized in the supporting file (S1 Table), providing a detailed overview for reference.

**Table 6:** Hierarchical regression results for ARPFG.

	ESE as the Moderator								
	Model 1		Model 2			Model 3			
Variable	β/t-value		SE	β/t-value		SE	β/t-value		SE
Age	.13^*^	2.03	.04	.14^*^	2.23	.04	.14^*^	2.19	.04
Gender	.15^**^	− 2.78	.05	−.14^**^	− 2.70	.04	−.14^**^	− 2.70	.04
Marriage	.01	.20	.01	.09	1.48	.09	.90	1.46	.05
Income	.07	1.30	.05	.06	1.05	.04	.05	.88	.04
Education level	.09	1.67	.08	.05	0.87	.08	.04	.81	.08
Religiosity	−.01	− .12	.06	−.03	− 0.51	.06	−.03	− .54	.06
MS				.28^***^	5.49	.04	.27^***^	5.51	.04
ESE				.19^**^	3.48	.05	.20^***^	3.74	.05
MS x ESE							−.12^*^	−2.34	.04
R^2^	.04			.15			.16		
ΔR^2^				.13^***^			.01^*^		

M1, Model 1 (control variables); M2, Model 2 (with control variables); M3, Model 3 (main model). ^*^p < .05, ^**^p < .01, ^***^p < .001. VIF ranges from 1-2.

APRFG =  Attitude towards plastic reduction for future generations; MS =  Mortality salience; ESE =  Explicit self-esteem (standardized Z-scores used)

**Table 7:** Result of conditional and conditional indirect effects.

Moderator	Condition effect of MS on APRFG	SE	t-value	P-value	LLCI		ULCI
-1SD ESE (3.97)	.34	.07	5.14	.0000	.2072		.4640
Mean ESE (4.84)	.24	.04	5.31	.0000	.1483		.3231
+1SD ESE (5.72)	.14	.05	2.54	.0116	.0305		.2411
Moderator	Conditional indirect effect of MS on Intention		Boot SE	BootLLCI		BootULCI	
-1SD ESE (3.97)	.18		.0363	.1065		.2480	
Mean ESE (4.84)	.12		.0252	.0750		.1739	
+1SD ESE (5.72)	.07		.0289	.0142		.1277	

Index of moderated mediation: -0.06 (Boot SE =.0241. BootLLCI & BootULCI [-.1087, -.0125])

MS =  Mortality salience; ESE =  Explicit self-esteem; APRFG =  Attitude towards plastic reduction for future generations; Intention =  Intention to reduce plastic use; LLCI, lower level confidence interval; ULCI, upper level confidence interval; SE, standard error.

### 4.3. Sensitivity analysis: consistency of effects with reduced sample size

To ensure the robustness of our findings, we conducted a sensitivity analysis by reducing the sample size. Given that the minimum sample size for this study is 272 and the existing sample size is 357, representing a difference of approximately 31%, we conducted sensitivity analysis by randomly removing up to 30% of the cases in SPSS. The results indicated that the moderation effect, mediation effect, and moderated mediation model all remained significant (p < .05). Also, when the sample size was reduced by 30%, the R² of the mediation model remained.60, which is close to the R² of.62 observed with 357 cases.

## 5. Discussion

This study explored whether attitude and intention toward plastic reduction, when considering future generations’ health, improve under the condition of MS. The findings showed that MS helps individuals with different explicit self-esteem levels to strengthen their APRFG, thereby positively influencing their plastic reduction intention.

The academic contributions of this study are as follows. Firstly, to the best of the authors’ knowledge, this study is among the first to use an experimental method to validate the relationship between MS and APRFG and intention to reduce plastic use. We induced MS by accentuating the environmental hazards of plastic products. The significant positive effects of MS on APRFG, subjective norm, perceived behavioral control, and plastic reduction intention are comparable to the effects of environmental concern identified in self-reported surveys by Paul et al. [37]. This indicates that MS, as an upstream factor influencing environmental concern [15,42], has a strong direct effect on APRFG and related measures.

Secondly, this study represents the first application of an integrated model of the Theory of Planned Behavior (TPB) and Terror Management Theory (TMT) to explore the factors influencing the intention to reduce plastic use. We found that planned attitudes and intentions can be implicitly influenced by MS. The existing literature on TMT suggests that MS might not directly affect green attitudes without mediators or moderators [14,15]. However, building on TMT literature indicating that MS favors attitude towards offspring, we discovered that at the distal stage, MS can directly influence both APRFG and intention to reduce plastic use. Based on TMT, we suggest that APRFG and plastic reduction intention to serve as a form of worldview defense that can enhance explicit self-esteem.

Thirdly, although explicit self-esteem is a common moderating variable in TMT, it is rarely used in conjunction with MS to explore plastic reduction attitude and intention. Literature indicates that individuals with low explicit self-esteem exhibit lower materialism [14], and high materialism weakens green consumption attitude and intention [13]. Therefore, individuals with low explicit self-esteem are more likely to embrace green consumption. This study further confirmed that under MS stimulation, individuals with lower explicit self-esteem are significantly more likely than those with higher explicit self-esteem to adopt a plastic reduction worldview for the benefit of future generations. Within TMT framework, this study validated potential causal relationships previously suggested by independent studies. Fourthly, APRFG, subjective norm, and perceived behavioral control fully mediated 87.5% of the relationship between MS and intention to reduce plastic use (indirect effect/ total effect), with the mediating effect of APRFG alone reaching 62%. This underscores the crucial role of APRFG. This study suggests that these three mediators induced by MS are key predictors of plastic reduction intention. Additionally, this moderated mediation model highlights the significant influence of explicit self-esteem and APRFG on the relationship between MS and intention to reduce plastic use, demonstrating that the connection between MS and plastic reduction intention involves several important explanatory variables, as supported by existing studies.

The managerial implications of this study are as follows. Firstly, based on our findings, we recommend leveraging the MS caused by the health hazards of plastics as a foundation for communicating plastic reduction strategies targeted at the public to consumers. For instance, emphasizing how microplastics harm health in promotional materials not only addresses the public’s knowledge needs but also directly enhances consumers’ attitude and intention towards plastic reduction. Secondly, since the health hazards of plastics can strengthen concerns for the well-being of future generations and improve attitude and intention to reduce plastic use, this information should be incorporated into educational materials for children and adolescents. Educating children and adolescents can enable them to convey the message to their parents. According to TMT [19], if attitude towards plastic reduction become a widely accepted and positive value, especially in collectivist societies, it can help reinforce in-group identity and promote long-term commitment to reducing plastic use. Thirdly, our study indicates that individuals with lower explicit self-esteem are a key target group for plastic reduction advocacy. Policymakers should focus on this demographic by creating communication materials that combine MS with messages about the benefits of reducing plastic use for future generations. This strategy is likely to significantly enhance their intention to reduce plastic use.

## 6. Limitation and future suggestions

This study has several limitations. Firstly, this research focused on a collectivist Eastern society, and national cultural values can influence how people respond to MS and their corresponding worldview defense behavioral intention [19,57]. Future researchers should replicate this model in individualistic cultures to assess its applicability. Secondly, this study used MS induced by highlighting the hazards of plastic as an intervention. However, besides the health hazards of plastics to humans, the impact of plastics on marine life [58] may be a more direct and meaningful intervention for countries that heavily rely on fisheries. Future researchers should consider selecting MS interventions suitable for the specific contexts of different countries. Thirdly, there may be other variables influencing the relationship between MS and behavioral intention of green consumption. Most TMT literature uses explicit self-esteem as the primary anxiety-buffering moderator. Future researchers should examine the relationship between implicit self-esteem and intention to reduce plastic use.

## Supporting information

S1 Appendix The text for the control group. (PDF)

S1 Table The empirical results of the hypotheses. (PDF)

S1 Dataset Experimental questionnaire results. (XLSX)
