# Supplementary material for: The impact of mortality salience and explicit self-esteem on plastic reduction intention: A moderated mediation model
Source: PLoS One. 2025 Mar 24;20(3):e0320059. doi: 10.1371/journal.pone.0320059 (PMC11932489; doi:10.1371/journal.pone.0320059)
Supplement: S1 Appendix — (PDF) [file pone.0320059.s001.pdf]

Toothache is often regarded as one of the most excruciating and relentless pains a person can endure. The throbbing sensation can feel like a hammer pounding inside your jaw, or sharp, piercing shocks radiating through your entire face. It often strikes unexpectedly, disrupting your ability to focus, eat, or even sleep. The pain can become so overwhelming that it consumes your thoughts, leaving you feeling powerless, frustrated, and desperate for relief.

Now, we would like you to recall a time when you experienced a severe toothache. Take a moment to reflect on how it felt and how it affected your daily life. Please describe the pain in 30 words or less—you can include what kind of pain it was, how intense it felt, or any thoughts or emotions you had during that time. Write down your feelings and experiences below.
