# Supplementary material for: The impact of mortality salience and explicit self-esteem on plastic reduction intention: A moderated mediation model
Source: PLoS One. 2025 Mar 24;20(3):e0320059. doi: 10.1371/journal.pone.0320059 (PMC11932489; doi:10.1371/journal.pone.0320059)
Supplement: S1 Table — (PDF) [file pone.0320059.s002.pdf]

The empirical results of the hypotheses

Hypotheses	Results
H1：MS is positively related to individuals' intention to reduce plastic use.	Supported
H2：MS is positively related to APRFG.	Supported
H3：MS is positively related to subjective norm.	Supported
H4：MS is positively related to perceived behavioral control.	Supported
H5：APRFG is positively related to individuals' intention to reduce plastic use.	Supported
H6：Subjective norm is positively related to individuals' intention to reduce plastic use.	Supported
H7：Perceived behavioral control is positively related to individuals' intention to reduce plastic use.	Supported
H8：APRFG mediates the relationship between MS and individuals' intention to reduce plastic use.	Supported
H9：Subjective norm mediates the relationship between MS and individuals' intention to reduce plastic use.	Supported
H10：Perceived behavioral control mediates the relationship between MS and individuals' intention to reduce plastic use.	Supported
H11：Explicit Self-esteem moderates the effect of MS on APRFG.	Supported
H12：Explicit Self-esteem moderates the indirect association between MS and individuals' intention to reduce plastic use via APRFG, such that the mortality salience-attitude pathway would be stronger in group with lower explicit self-esteem.	Supported
